# Neurophysiological Progression in Alzheimer's Disease: Insights From Dynamic Causal Modelling of Longitudinal Magnetoencephalography

**DOI:** 10.1002/hbm.70234

**Published:** 2025-05-21

**Authors:** Amirhossein Jafarian, Melek Karadag Assem, Ece Kocagoncu, Juliette H. Lanskey, Haddy Fye, Rebecca Williams, Andrew J. Quinn, Jemma Pitt, Vanessa Raymont, Stephen Lowe, Krish D. Singh, Mark Woolrich, Anna C. Nobre, Richard N. Henson, Karl J. Friston, James B. Rowe

**Affiliations:** ^1^ MRC Cognition and Brain Sciences University of Cambridge Cambridge UK; ^2^ Department of Clinical Neurosciences and Cambridge University Hospitals NHS Foundation Trust Cambridge Biomedical Campus Cambridge UK; ^3^ Oxford Centre for Human Brain Activity, Wellcome Centre for Integrative Neuroimaging, Department of Psychiatry University of Oxford Oxford UK; ^4^ Department of Psychology University of Birmingham Birmingham UK; ^5^ Department of Psychiatry University of Oxford Oxford UK; ^6^ Lilly Centre for Clinical Pharmacology Singapore; ^7^ Cardiff University Brain Research Imaging Centre, School of Psychology Cardiff University Cardiff UK; ^8^ Department of Psychology and Centre for Neurocognition and Behaviour Wu Tsai Institute, Yale University New Haven Connecticut USA; ^9^ Queen Square, Institute of Neurology University College London London UK

**Keywords:** Alzheimer's disease, dynamic causal modelling, magnetoencephalography, parametric empirical Bayes

## Abstract

Neurodegenerative diseases, including Alzheimer's disease, are characterised by selective neuronal vulnerability with regional, laminar, cellular and neurotransmitter specificity. The regional losses of neurons and their synapses are associated with neurophysiological changes and cognitive decline. Hypotheses related to these mechanisms can be tested and compared by dynamic causal modelling (DCM) of human neuroimaging data, including magnetoencephalography (MEG). In this paper, we use DCM of cross‐spectral densities to model changes between baseline and follow‐up data in cortical regions of the default mode network, to characterise longitudinal changes in cortical microcircuits and their connectivity underlying resting‐state MEG. Twenty‐nine people with amyloid‐positive mild cognitive impairment and Alzheimer's disease early dementia were studied at baseline and after an average interval of 16 months. To study longitudinal changes induced by Alzheimer's disease, we evaluate three complementary sets of DCM: (i) with regional specificity, of the contributions of neurons to measurements to accommodate regional variability in disease burden; (ii) with dual parameterisation of excitatory neurotransmission, motivated by preclinical and clinical evidence of distinct effects of disease on AMPA versus NMDA type glutamate receptors; and (iii) with constraints to test specific clinical hypothesis about the effects of disease‐progression. Bayesian model selection at the group level confirmed evidence for regional specificity of the effects of Alzheimer's disease, with evidence for selective changes in NMDA neurotransmission, and progressive changes in connectivity within and between Precuneus and medial prefrontal cortex. Moreover, alterations in effective connectivity vary in accordance with individual differences in cognitive decline during follow‐up. These applications of DCM enrich the mechanistic understanding of the pathophysiology of human Alzheimer's disease and inform experimental medicine studies of novel therapies. More generally, longitudinal DCM provides a potential platform for natural history and interventional studies of neurodegenerative and neuropsychiatric diseases, with selective neuronal vulnerability.

## Graphical abstract

We employ dynamic causal modeling (DCM) of resting‐state MEG data to model Alzheimer's disease (AD)‐related changes in cortical microcircuits and connectivity within the default mode network over 1‐year duration. Our DCM incorporates region‐specific contributions of cortical laminar activities to accommodate variability in disease burden across brain regions. We introduce dual parameterization of neurotransmission mechanisms in DCM to distinguish between AD's effects on different receptor types. Bayesian group‐level analysis reveals regional specificity of AD effects, highlighting selective NMDA neurotransmission changes and progressive connectivity alterations within and between the precuneus and medial prefrontal cortex. Changes in effective connectivity correlate with individual differences in cognitive decline during follow‐up, suggesting potential as a biomarker for AD progression.

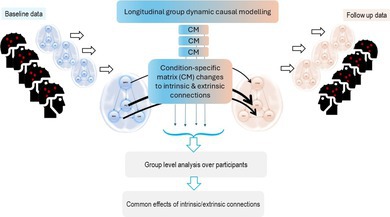

## 1 Introduction

Neurodegenerative diseases are characterized by selective neuronal vulnerability leading to pathophysiology with regional, laminar, cellular, and neurotransmitter specificity. For example, Alzheimer's disease (AD) induces tau‐ and amyloid‐aggregates, synaptopathy, and cell loss in a well‐characterized spatiotemporal order. This regional pattern of progression underlies the Braak staging of disease (Braak and Braak 1997). In contrast, each of the syndromes of frontotemporal lobar degeneration is associated with selective regional pathophysiology in prefrontal, temporal, and insular regions (Neary et al. 1998; Pievani et al. 2011). The regional loss of synapses in AD and frontotemporal dementia is associated with cognitive decline (Holland et al. 2023; Terry et al. 1991; Malpetti et al. 2024) and corresponds to changes in neurophysiological function as measured by electro−/magneto‐encephalography (E/MEG) (Sami et al. 2018; Kocagoncu et al. 2022). AD and frontotemporal dementias also have relatively selective effects on the neurotransmitter systems, such as GABA, glutamate, noradrenaline, and acetylcholine (Carello‐Collar et al. 2023; Murley and Rowe 2018; Holland et al. 2023). The reasons for this selectivity are poorly understood. However, non‐invasive in vivo localization and quantification of selective neuronal deficits, below the resolution of structural brain imaging (e.g., MRI), would facilitate mechanistic characterization of disease and accelerate experimental medicine studies (Shaw et al. 2020; Adams, Hughes, et al. 2021; Jafarian et al. 2023; Pinotsis et al. 2016).

The effect of AD on synaptic function can be linked to direct synaptopathy of oligomeric tau and beta‐amyloid (Guerrero‐Muñoz et al. 2015) and indirect synaptopathy from microglial‐mediated neuroinflammation (Henstridge et al. 2019; Tzioras et al. 2023). Synaptopathy precedes the loss of cell viability and cell death (Grierson et al. 2022; Buccarello et al. 2017; Schneider and Mandelkow 2008; Sperling et al. 2011). Before the loss of activity and connectivity in late‐stage disease, a period of transient neuronal hyper‐excitability and hyper‐connectivity has been observed (Koelewijn et al. 2019; Lanskey et al. 2024; Pusil et al. 2019; Bajo et al. 2012; López et al. 2014), and a dysregulation of the excitatory‐inhibitory balance controlling induced and oscillatory dynamics (Reinders et al. 2016). These pathophysiological changes underlie cognitive and behavioral deficits and can be measured non‐invasively from electro‐ and magnetoencephalography. In mild or moderate stages, Alzheimer's disease changes the evoked and spectral features of EEG and MEG (Babiloni et al. 2016; Moretti et al. 2004; Yener and Başar 2010, 2013). Data‐driven, biophysically‐informed generative models, in particular dynamic causal modeling of such non‐invasive recordings, can identify specific effects of disease by region, layer, cell type, and class of receptor or ion channels (e.g., Jafarian et al. 2024, 2023; Adams, Pinotsis, et al. 2021; Cope et al. 2022; Shaw et al. 2021; Gilbert et al. 2016; Symmonds et al. 2018), and potentially facilitate precision medicine studies.

To address the broader context of computational modelling approaches in neurophysiology, we acknowledge several prominent platforms alongside Dynamic Causal Modelling (DCM) (Friston et al. 2003), including The Virtual Brain (TVB) (Jirsa et al. 2023, 2014), Human Neocortical Neurosolver (HNN) (Neymotin et al. 2020), and the Blue Brain Project platform (Markram 2006). While each offers unique strengths—such as TVB's proficiency in brain‐wide network modelling and epilepsy research, HNN's specialisation in single‐source MEG data modelling, and Blue Brain's high biophysical details—DCM was chosen for this study due to its robust model comparison capabilities, flexibility across neuroimaging modalities, and balance between biological plausibility and computational efficiency. DCM's ability to integrate theoretical models with empirical data for direct hypothesis testing, coupled with its capacity to model both small‐scale circuits and larger networks, makes it particularly suited for our translational modelling approach and foundational questions that arise in experimental medicine. However, we recognise the ongoing development in this dynamic field and the potential of alternative approaches for different research questions.

In this study, we aimed to quantify how Alzheimer's disease changes the regional, cellular and neurotransmitter components of the cortical generators of MEG, via DCM (Jafarian et al. 2024). We sought to quantify not only baseline effects of Alzheimer's disease but also the progression within subjects over time. We study the default mode network (DMN), which is widely used to characterize changes in task‐free functional neuroimaging data. Resting state MEG is well‐tolerated and sensitive to the presence, severity, and progression of AD in terms of its spectral correlates (Schoonhoven et al. 2022; Poil et al. 2013). Resting‐state MEG protocols can be readily and safely repeated, making them suitable for longitudinal and interventional studies (Stam 2010).

For longitudinal‐DCM of Alzheimer's disease, we need to consider three variations of DCM. First, we distinguish the effects of disease on AMPA (α‐amino‐3‐hydroxy‐5‐methyl‐4‐isoxazolepropionic acid) versus NMDA (N‐methyl‐D‐aspartate) receptors (Armstrong et al. 1994; Pickard et al. 2000), replacing the single glutamatergic parameter in current (canonical) neural mass models. Alterations in AMPA and NMDA receptors in superficial and deep layers of the cortex affect cognition (Yasuda et al. 1995; Myme et al. 2003). Crucially, AD may have differential effects on NMDA and AMPA receptors (Ikonomovic et al. 1995): a hypothesis we test by comparing the evidence for models (i.e., Bayesian model comparison) with and without separate glutamatergic receptor subtypes.

Second, we introduce regional inhomogeneity into the contributions of each cell class to the observed spectral density. Usually, DCM of spectral responses assumes that the contribution of neuronal populations to local field potentials is conserved over regions, modeling only regional variations in sensor gain. However, the regional variation and progression of AD are likely to require models that permit heterogeneity in the contribution of hidden neuronal states: a hypothesis that we test by comparing models with and without regional heterogeneity.

Third, we model the progression of Alzheimer's disease at the neuronal level using condition‐specific parameters. Crucially, clinical information is used to specify hypotheses about how the disease affects a subset of neuronal mechanisms between baseline and follow‐up for example, (Seoane et al. 2024). This enriches and complements current findings based on functional data analysis (e.g., Schoonhoven et al. 2022, 2023).

Given a “winning” model (i.e., the model with highest evidence amongst other models), we then test whether the neuronal mechanisms underlying AD progression are associated with cognitive decline. Embedding clinical severity scores within the DCM enables one to test their associated underlying neuronal influences. Collectively, our approach allows comparison of hypotheses about between‐group, between‐severity, and between‐time differences. This is a version of the use of empirical priors to improve the model evidence under Bayesian inversion (Hinton and van Camp 1993).

We evaluate these alternative modeling approaches in the context of longitudinal resting‐state MEG from people with symptomatic amnestic Alzheimer's disease (Lanskey et al. 2022). Specifically, we identify the causes of changes in MEG spectral density between baseline (BL) and annual follow‐up (AF). We set out and tested specific hypotheses regarding the effects of Alzheimer's disease on AMPA and NMDA receptor‐mediated synaptic transmission, regional heterogeneity within default mode network nodes (precuneus, medial prefrontal cortex, angular gyri), and longitudinal changes across disease phases. These hypotheses were evaluated using Bayesian model selection within the DCM framework to elucidate the mechanistic underpinnings of AD progression, as explained in the Methods section. We use Bayesian model selection to identify the most likely explanation for longitudinal spectral changes in MEG, as a function of synapse type, cell class, cortical layer, and region‐specific effects of disease. We discuss the potential applications and limitations of the method, not only to understand AD pathophysiology, but also other disorders and therapeutic interventions. Glossary and definitions of acronyms and variables used in this paper are provided in Tables 1, 2 and 3, and the space of hypotheses associated with AD progression is listed in Table 4.

**TABLE 1:** Acronyms.

Acronyms	Description
AD	Alzheimer's disease
AF	Annual follow up MEG data
AMPA	α‐amino‐3‐hydroxy‐5‐methyl‐4‐isoxazolepropionic acid
ACE‐R	Addenbrooke's Cognitive Examination Revised
BL	Baseline MEG data
BMR	Bayesian model reduction
CMM	Conductance microcircuit model
DCM	Dynamic causal modelling
ERP	Event related potential
FT	Fourier transform
fMRI	Functional magnetic resonance imaging
GABA	Gamma‐aminobutyric acid
GLU	Glutamate
MEG	Magnetoencephalography
MRS	Magnetic resonance spectroscopy
NMDA	N‐methyl‐D‐aspartate receptors
CM	Condition‐specific matrix
PEB	Parametric empirical Bayes
PET	Positron emission topography
PSDs	Power spectral density that was derived from MEG data
PiB	Pittsburgh Compound‐B
STL	State‐to‐lead field
SP, SS, Inh, DP	Superficial pyramidal cells, spiny stellate excitatory neurons, interneurons, deep pyramidal cells

## 2 Material and Methods

### 2.1 Participants

The New Therapeutics in Alzheimer's Disease (NTAD) study had ethical approval from the Cambridge Central Research Ethics Committee, East of England. Participants provided written informed consent prior to engagement with any study activities. Participants whose data is included in this analysis had symptomatic Alzheimer's disease, with positive amyloid biomarkers and episodic memory deficits (Lanskey et al. 2022), either as mild cognitive impairment or early dementia. Participants with symptomatic AD had either mild cognitive impairment or early dementia according to clinical diagnostic criteria (Albert et al. 2011) or (Mckhann et al. 2011) and all had positive biomarkers for AD pathology with either positive Pittsburgh Compound‐B (PiB) amyloid PET scan and/or positive cerebrospinal fluid biomarkers (Lanskey et al. 2022).

Forty‐five participants completed baseline resting state MEG sessions, of whom twenty‐nine completed follow‐up scanning. The study intended a 12‐month follow‐up, typical of the duration of many early phase clinical trials, but this was extended to 16 months due to the COVID19 pandemic (Lanskey et al. 2022). Participants' mean age was 74 (±7.67 std), mini‐mental state examination score 25.4 (±3.1 std., MMSE max 30 points), and revised Addenbrooke's Cognitive Examination score 75.6 (±10.2 std., ACER max 100 points). All participants were biomarker‐positive for beta‐amyloid by either PiB PET imaging or CSF examination.

#### 2.1.1 Resting State MEG Data

Participants underwent 5 min of resting‐state MEG with eyes open using an Elekta Vector View system with 204 planar gradiometers and 102 magnetometers with 1000 Hz sampling rates. Bipolar electro‐oculogram and electrocardiogram electrodes were employed to record participants' horizontal and vertical eye movements. To track the head position, the EEG cap was equipped with five head location indicator coils and Polhemus digitisation with three fiducial points (nasion, left and right pre‐auricular) and > 100 head shape points were used for digitisation. The MaxFilter v2.2.12, Elekta Neuromag toolbox (Elekta Oy) was used for detection and interpolation of bad sensors, temporal signal space separation, noise removal from the data, and corrections of head movements. The pre‐processing of MEG data included down‐sampling to 500 Hz, a bandpass filter between 0.1 and 100 Hz, and a Notch filter between 48–52 Hz and 98–102 Hz, followed by OSL toolbox‐based artefact rejection/removal using ICA, with EOG data. We epoch the data into 1‐s segments, with artefact rejection and removal on each segment.

We chose to study eyes‐open resting state data for several reasons. Firstly, in our patient cohort, eyes‐open recordings provided superior data quality and lower risk of confounds such as participants inadvertently falling asleep. Secondly, we employed advanced artifact removal techniques (OSL‐ICA) to mitigate potential increased artifacts in eyes‐open conditions. Thirdly, eyes‐open resting states arguably more closely resemble everyday awake resting states, enhancing the ecological validity of our findings. There are typically variations in alpha activity between eyes open and closed; however, our DCM study focuses on modeling of the wide frequency spectrum, not merely narrow band power.

T_1_‐weighted structural MRI (3 T Siemens MPRAGE, TR = 2300 ms, TE = 2.91 ms, resolution 1 mm) of each participant, underwent DICOM conversion to Nifti format. We used SPM12's canonical cortical mesh of medium resolution (~4000 vertices per hemisphere), plus skull and scalp meshes (~2000 vertices total), to inverse‐normalise from MNI space to match each individual's native MRI. Using three fiducials and head shape points, MRI data were co‐registered with the scalp mesh, and the cortical and inner‐skull meshes used to create a single‐shell boundary element forward model of MEG. Inversion of currents, oriented perpendicular to the local curvature at each cortical vertex, was estimated using SPM12's empirical Bayesian framework, with the “COH” option for regularisation, encouraging smooth solutions (Litvak et al. 2011). The MEG data used for inversion were restricted to the frequency range 0.1–100 Hz across all segments, and the magnetometer and gradiometer data were fused (Henson et al. 2011). The estimated source time‐courses where then extracted for four default mode network sources: left and right angular gyri (LAG [49–63 33], RAG [−46–66 30]), medial prefrontal cortex (MPFC) [−1 54 27], and Precuneus (PCC) [0–55 32]. Note that we chose this two‐step approach of distributed inversion of all potential cortical sources followed by applying DCM to the activity estimated from our DMN sources (treating the data as if we had recorded LFPs directly). In effect, crosstalk in estimating their activity from the sensor data is small.

To characterise the interactions between the selected DMN regions, DCM used cross‐spectral density (CSD) as the data feature, calculated within and between all pairs of sources. An 8th‐order autoregressive model is used to model the PSD of each trial (1 s duration here) within and between sources. Then the first principal component of these PSDs across trials was used as the data feature for DCM.

### 2.2 DCM of Resting State MEG Data

DCM is Bayesian inversion of neuronal models from features of neuroimaging data, using the variational Free energy (a lower bound on log‐model evidence, i.e., model accuracy minus complexity). The evidence for alternative hypotheses—represented by alternative models—is compared through the Bayesian model selection procedure to identify the most likely explanation for the data (Kass and Raftery 1995; Friston 2011; Friston et al. 2011; Jafarian et al. 2019). Bayesian model reduction (BMR) enables the comparison over an extensive model space by comparing a full model with reduced models, formed by removing parameters (Friston and Penny 2011; Friston et al. 2019). At the group level, parametric empirical Bayesian (PEB) takes into account all first‐level (single subject) DCMs to accommodate random effects of participant and/or session at the group level (Friston et al. 2015, 2016; Litvak et al. 2015). This hierarchical modelling approach has been validated in a range of cross‐sectional pharmacological, autoimmune, and genetic models for example, (Symmonds et al. 2018; Adams, Hughes, et al. 2021; Cope et al. 2022; Jafarian et al. 2023, 2024, 2020; Gilbert et al. 2016).

**FIGURE 1:**
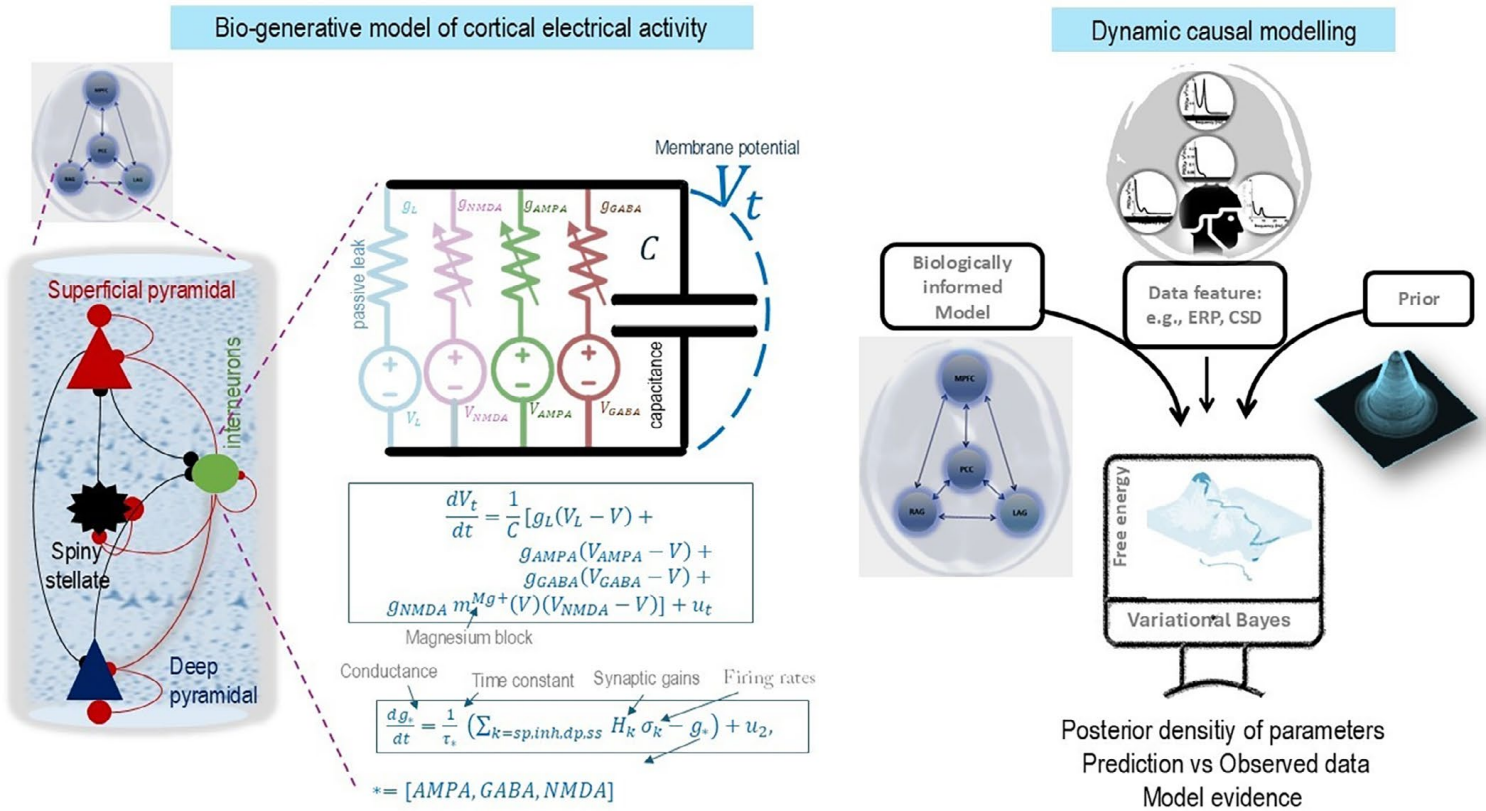
Dynamic Causal Modelling (DCM) of neuroimaging data features. Right panel: This graphic illustrates the model, data feature, and variational Bayesian procedures of the DCM approach. In DCM, the evidence lower bound cost function with respect to model parameters is optimised using a variational Bayesian scheme to infer biologically informed model parameters (e.g., synaptic efficiency) and model evidence based on empirical neuroimaging data features (e.g., event related potential or power spectral density of MEG data). The left panel shows a model of a cortical column (i.e., node) with four populations: Excitatory spiny cells, interneurons, and superficial and deep pyramidal cells. The dynamics of each population are governed by the Morris‐Lecar conductance‐based model (Moran et al., 2013). This model describes the interactions among membrane potentials, ion conductance, and firing rates. Nodes or sources in the model are connected via forward and backward connections.

We use DCM for cross‐spectral density (CSD) (Friston et al. 2012) as summarised in Figure 1 and explained in more detail in Appendix A. Our objective was to establish a framework for characterising the common neurophysiological mechanisms underlying AD progression. By focusing on shared patterns, we aimed to identify reliable targets for intervention that could benefit a broad range of patients. Individual variability also occurs in AD, and the DCM framework can be extended to address this. For example: (I) DCM parameters can be used to stratify patients into subgroups based on their unique neurophysiological profiles, potentially informing personalized treatment strategies, (II) longitudinal DCM data can be used to predict individual trajectories of cognitive decline, allowing for more targeted interventions at specific disease stages, and (III) individual DCM parameters can be used to predict a patient's likelihood of responding to a particular therapy, enabling personalized treatment selection. While our current study focuses on identifying common neurophysiological mechanisms underlying AD progression, we acknowledge the potential value of individual variability in disease trajectories. Future work can address individual trajectories of cognitive decline and predict treatment response, advancing the goal of precision medicine.

**TABLE 2:** Glossary of variables and expressions in the conductance‐based model (CMM‐NMDA).

Variable	Description
$u_{t}$	Exogenous input
$V_{t}$	Mean depolarisation of a neuronal population
$\sigma \left(v\right)$	The neuronal firing rate—a sigmoid squashing function of depolarisation
L	Lead field vector mapping from (neuronal) states to measured (electrophysiological) responses

**TABLE 3:** Parameters of the neuronal model (see also Figure 1). The $\left(i.j\right)$ element in the matrix associated with parametrisation of intrinsic connection H, means connections that originate from population j and target population i in a region (here element 1 to 4 are corresponds to ss, sp., inh and dp layers respectively). The $\left(i.j\right)$ element in the matrix associated with parametrisation of extrinsic connections A and AN, means connections that originate from population j in a region and project to population i in a distal region (here element 1 to 4 are corresponds to ss, sp., inh and dp layers respectively).

	Description	Parameterisation	Prior
κ	Rate constants of ion channels, AMPA, GABA, and NMDA respectively.	$\exp\left(\theta_{\kappa }\right)\cdot \kappa$ $\kappa =\left[4,16,100\right]$	$p\left(\theta_{\kappa }\right)=N\left(0,1/16\right)$
C	Membrane capacitance of SS, Sp, Inh, and Dp populations respectively.	$\exp\left(\theta_{c}\right)\cdot C$ $C=\left[128\;128\;256\;32\right]/1000$	$p\left(\theta_{c}\right)=N\left(0,1/16\right)$
H	Intrinsic connections in default DCM.	$\exp\left(\theta_{H}\right)\cdot H$ $H=\left[\begin{array}{c} 8\;0\;2\;0\\ \;4\;8\;2\;0\;\\ 4\;0\;32\;2\\ 0\;4\;8\;128 \end{array}\right]$	$p\left(\theta_{H}\right)=N\left(0,1/32\right)$
$H_{i}$	Intrinsic inhibitory connections in NMDA/AMPA separated DCM.	$\exp\left(\theta_{H_{i}}\right)\cdot H_{i}$ $H_{i}=\left[\begin{array}{c} 8\;0\;2\;0\\ \;0\;8\;2\;0\;\\ 0\;0\;32\;0\\ 0\;0\;8\;128 \end{array}\right]$	$p\left(\theta_{H_{i}}\right)=N\left(0,1/32\right)$
$H_{\text{nmda}/\text{ampa}}$	Intrinsic NMDA/AMPA connections in NMDA/AMPA separated DCM.	$\exp\left(\theta_{H_{\mathrm{nma}/\text{ampa}}}\right)\cdot H_{\text{nmda}/\text{ampa}}$ $H_{\text{nmda}/\text{ampa}}=\left[\begin{array}{c} 0\;0\;0\;0\\ 4\;0\;0\;0\;\\ 4\;0\;0\;2\\ \;0\;4\;0\;0 \end{array}\right]$	$p\left(\theta_{H}\right)=N\left(0,1/32\right)$
A	Extrinsic forward connection.	$\exp\left(\theta_{A}\right)\cdot A$ *A* = [1 0; 0 1; 0 2; 0 0]/8	$p\left(\theta_{A}\right)=N\left(0,1/8\right)$
AN	Extrinsic backward connection	$\exp\left(\theta_{\text{AN}}\right)\cdot \text{AN}$ AN = [1 0; 0 1; 0 2; 0 0]/8	$p\left(\theta_{A}\right)=N\left(0,1/8\right)$
L	Sensor gain or lead field	L	$p\left(L\right)=N\left(1,64\right)$
J	Contribution of spiny stellate population ($J_{\mathrm{ss}}$) and deep pyramidal ($J_{\mathrm{dp}}$) to observation data. In modified DCM, region‐specific J's with the same prior as default DCM is considered.	$J_{*}$, (*=dp,ss)	$p\left(J_{\mathrm{ss}}\right)=N\left(0,1/16\right)$ $p\left(J_{\mathrm{dp}}\right)=N\left(0,1/16\right)$
a	Endogenous random fluctuation with transfer function $\frac{a_{1}}{\omega^{a_{2}}}$.	$\exp\left(a\right)$	$p\left(a_{1,2}\right)=N\left(0,1/128\right)$
d	Structural cosine coefficients of endogenous random fluctuation.	$\exp\left(d\right)$	$p\left(d_{1,2,3,4}\right)=N\left(0,1/128\right)$
b	Common sensor noise with transfer function $\frac{b_{1}}{\omega^{b_{2}}}$.	$\exp\left(b\right)$	$p\left(b_{1,2}\right)=N\left(0,1/128\right)$
c	Specific sensor noise with transfer function $\frac{c_{1}}{\omega^{c_{2}}}$.	$\exp\left(c\right)$	$p\left(c_{1,2}\right)=N\left(0,1/128\right)$
f	Scaling some frequencies as model of data filtration.	$\exp\left(f\right)$	$p\left(f_{1,2}\right)=N\left(0,1/128\right)$
D	Delay between regions and within layers.	$\exp\left(D\right).D$ $D=\left[2,16\right]$	$p\left(D\right)=N\left(0,1/64\right)$

We evaluate three variations to the standard DCM approach as what follows with space of all hypotheses in this paper is given in Table 4 to model progression of AD.

**TABLE 4:** Hypotheses space associated with AD progression.

Receptor‐specific hypotheses	
Hypothesis 1	Alzheimer's disease progression preferentially affects NMDA and AMPA receptor‐mediated synaptic transmission, consistent with preclinical evidence of differential receptor vulnerability.
Region‐specific hypotheses	
Hypothesis 2	The Precuneus and medial prefrontal cortex (key nodes of the default mode network) show greater synaptic dysfunction compared to other regions of DMN (e.g., angular gyri), due to their early vulnerability in AD.
Hypothesis 3	Regional heterogeneity in synaptic dysfunction correlates with regional amyloid burden or tau pathology, as suggested by prior imaging studies.
Disease‐phase hypotheses	
Hypothesis 4	In early stages of AD, there is transient hyperexcitability and hyperconnectivity within the default mode network, followed by progressive hypoactivity and reduced connectivity as the disease advances.

#### 2.2.1 Separation of Glutamatergic NMDA and AMPA Parameters

First, we re‐parameterize DCM to distinguish AMPA (α‐amino‐3‐hydroxy‐5‐methyl‐4‐isoxazolepropionic acid) and NMDA (N‐methyl‐D‐aspartate) related parameters.

##### 2.2.1.1 Motivation

In Alzheimer's disease (AD), changes in the expression and function of AMPA and NMDA receptors can occur throughout the cerebral cortex, and the severity of these changes may vary across different cortical layers and regions. Specifically, changes in AMPA and NMDA receptor expression in layer II neurons have been reported in AD, contributing to altered synaptic transmission and impaired connectivity (Domínguez‐Álvaro et al. 2021). In layer III, neurons are involved in corticocortical connections, and disruptions in AMPA and NMDA receptor functions can affect communication between cortical regions and contribute to cognitive deficits in AD. Alterations of AMPA and NMDA receptors in layer V neurons can impact corticofugal projections and contribute to motor and cognitive dysfunction in AD. Here, we limit ourselves to cortical regions due to their sensitivity in MEG data. Alterations in AMPA and NMDA receptor expression and function in the hippocampus can also change synaptic function, plasticity, and memory.

##### 2.2.1.2 Implementation

In DCM conductance‐based models, AMPA and NMDA receptor‐mediated effects are parameterised with a single set of synaptic parameters. To test hypotheses about selective patterns and severity of changes in AMPA and NMDA receptors—across layers and cortical regions—we re‐parametrised a conductance‐based DCM to include both kinds of excitatory parameters. In short, we consider separate parameters for AMPA and NMDA connections (with priors comparable to the default excitatory parameters in DCM) and test whether such a modification improves model evidence, given the MEG data from our cohort.

#### 2.2.2 DCM With Region‐Specific State‐to‐Lead Fields

In conventional DCM for MEG, a single set of parameters is used to model the state‐to‐lead field (STL) gain across all regions. We relaxed this prior constraint to allow regional variation in the STL.

##### 2.2.2.1 Motivation

As AD progresses, different brain regions experience varying degrees of vulnerability, atrophy, and synaptopathy (e.g., disconnections from other regions and reduced activity), with regional variation in the layer‐specific contributions to neural activity. A discrepancy in activity levels of the regions (e.g., lower activity of PCC compared to left angular gyrus) could lead to reduced sensitivity of the likelihood function to the changes in regions with lower activity (Jafarian et al. 2024). However, from a clinical perspective, understanding the mechanisms of these disease‐related sources is crucial for answering important questions about AD progression and its impact on neural dynamics. In short, this re‐parameterisation departs from default DCM models, which assume the STL parameters are conserved over regions (Friston et al. 2012). Our hypothesis was that this re‐parameterisation increases the model evidence, implying that the relative density of neuronal cell types varies over regions. Specifically, this new parameterisation makes the likelihood function sensitive to sources whose mean field contribution to the measured signals is smaller than other sources (e.g., the measurable power of PCC sources is much smaller than other sources, in AD patients).

##### 2.2.2.2 Implementation

STL parameters control the degree to which the activity of specific layers contributes to the electrophysiological data. By allowing these to vary across regions, one can, in principle, increase the sensitivity of DCM to subtle differences which may be important to capture clinically. Therefore, we model separate STL parameters for each region, with the same prior as the single STL parameter in default applications of DCM. In each region, the activity of the superficial layer contributes directly to the local field potential; however, the contributions of the deep layer and layer IV neurons are estimated from data. In other words, the estimation allows for region‐specific estimation of relative weights of deep and layer IV contributions to local field potentials.

#### 2.2.3 DCM With Modified Condition Specific Matrix

**FIGURE 2:**
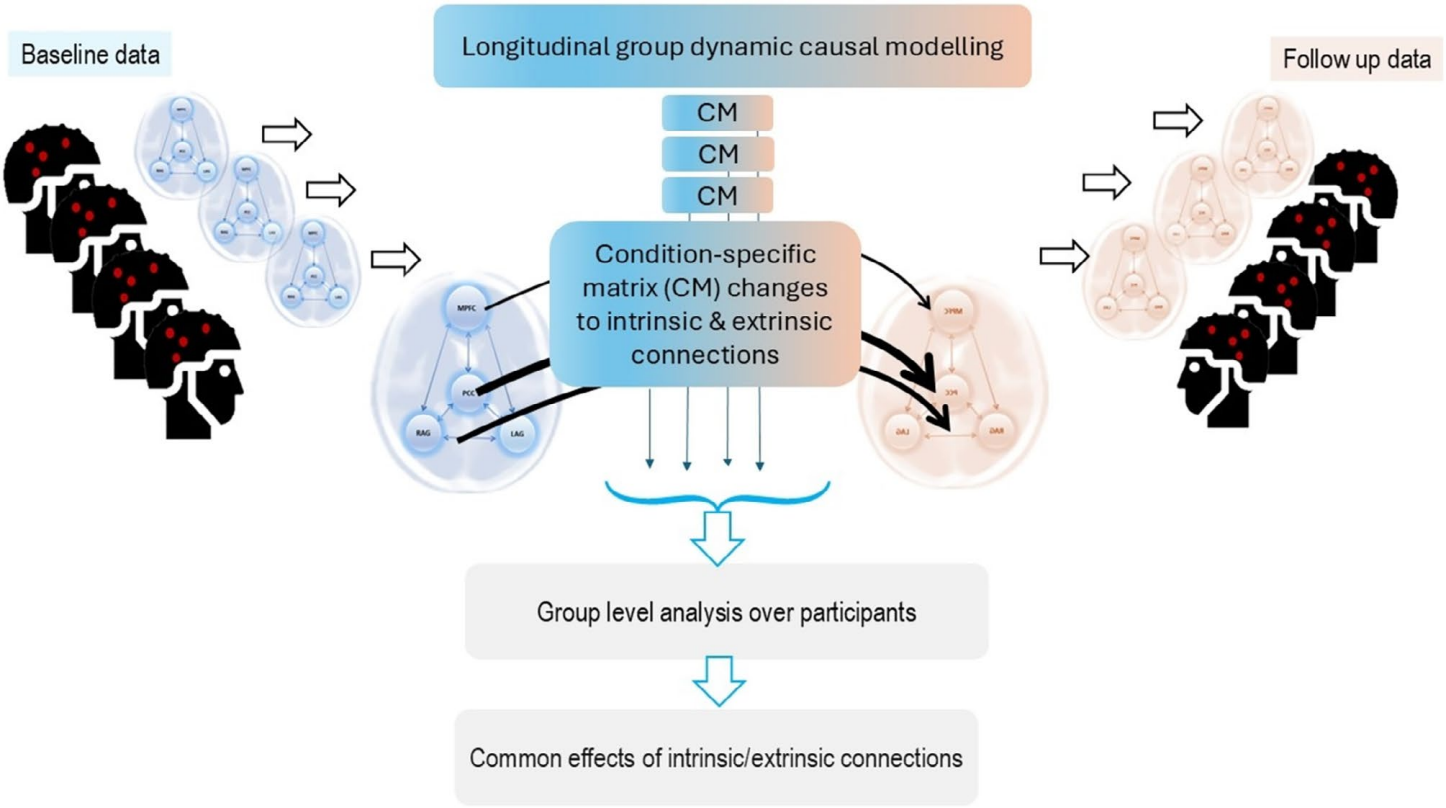
Modelling AD progression using longitudinal DCM. The aim is to find an optimal condition‐specific matrix (CM) that maximizes the group DCM model evidence. Each individual DCM models the BL data and replicates the AF data by perturbing the CM parameters, that is, morphing BL to AF is achieved by altering parameters of BL by CM. In this paper, we explore group‐level model comparison between noninformative CM and where clinical information informs CM and selects likely ones by Bayesian model selection. In the second‐level model, the design matrix is akin to a general linear model formulation, where the first column is set to one and the other column(s) contain one or more types of empirical information. One can then assess the impact of the empirical priors (and their interactions) by comparing free energy.

We aim to test hypotheses about the progression of Alzheimer's disease at the neuronal level by including markers of individual differences associated with AD progression. These markers could, in principle, be molecular, genetic, cognitive scores, or other modalities. The generic form of our implementation is illustrated in Figure 2, which leverages empirical priors for modeling differences between baseline and annual follow‐up at the neuronal level using a condition‐specific matrix (CM).

##### 2.2.3.1 Motivation

In DCM, unknown condition‐specific parameters are introduced as additive perturbations to parameters associated with one condition (e.g., $\theta_{\mathrm{BL}}$) to replicate another ($\theta_{\mathrm{AF}}$). The notation for condition‐specific matrices (CM) in the DCM community is B. Akin to the parameterisation of neuronal variables in DCM (see Table 2), where parameters are determined by the multiplication of default values (e.g., synaptic gains) and their data‐driven estimates, the parameterisation of B variable is given by XB. Conventionally, non‐informative categorical parameterisation (e.g., unity for X) is used to model one condition with respect to another where are neuronal parameters are given equal chance to explain between condition effects (Jafarian et al. 2019; Friston 2011; Friston et al. 2003). However, parameterisations in which only few neuronal mechanisms are allowed to be changed can also be useful to test hypotheses about the effects of disease progression in a nonlinear system.

In the context of AD progression, where the involvement of one brain region may be different to others (including the possibility that the disease does not affect some regions), the form of X is not known a priori. We therefore propose alternative hypotheses about the form of the X matrix and determine which one is more likely, via Bayesian model comparison.

We leverage clinical information about AD progression for specifying X (i.e., to sparse hypothesis space) and subsequently for inference of the unknown condition‐specific parameters B. Such non‐trivial definition of X in the context translational modelling allows to elucidate details neuronal mechanisms underpin complex effects of AD progression.

We first estimate subjects' individual DCMs and then assess the commonalities among ensuing B matrices over the group to reveal the likely pattern of AD progression, please see Figure 2 for details.

##### 2.2.3.2 Implementation

We use known clinical information about AD as the information to postulate different hypotheses about parameterisation of X. We are then performed neuronal inversion of BL versus AF having used clinical knowledge to specify the form of X and subsequently precision translational modelling of AD progression.

The mathematical formulation of condition specific stage in the DCM that links annual follow‐up data and baseline data reads as follows:

$$\theta_{\mathrm{BL}}+\mathrm{XB}\to \theta_{\mathrm{AF}}$$

**FIGURE 3:**
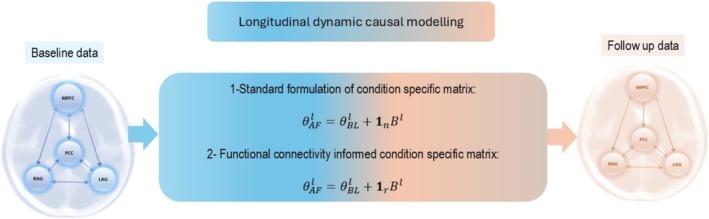
Longitudinal DCM can incorporate individualised differences, and connection‐specific effects of disease progression, using condition‐specific matrices (CM). We assess two options to model annual follow‐up data (AF) of a participant l by applying CMs to their inferred baseline data (BL) parameters. The first option is the default ‘non‐informative’ design which assumes that adding CM (with represent changes in n connections) to $\theta_{\mathrm{BL}}$ replicates the AF data. The second option allows only a subset of r<n connections to change as a result of AD progression (the value of *X* for other connections is fixed at 0). The selection of which connections are changing is informed by clinical information.

The condition‐specific matrix XB is the element‐wise product of pre‐specified sensitivity—or modulation—X and unknown parameters B that are inferred by DCM from empirical data. To model progression of AD from resting states MEG, we leverage inclusion of prior information about which regional neuronal mechanisms are likely to change over time. Figure 3 shows the different parameterisations for X options that we used in this paper:

1. Standard condition‐specific matrix: The longitudinal pathophysiology of AD is modelled using DCM with a standard CM equal to the unity matrix in equation 1 (option 1 for CM in Figure 3). This enables the modification of synaptic physiology between baseline and follow‐up data without making specific assumptions about the nonlinear nature of disease progression (i.e., some regions and/or between regions connections may be more affected by the disease). This approach can be viewed as non‐informative prior assumption about the disease progression. In this approach morphing one conditions to others is merely driven by the model likelihood. In other words, only the data lead to adjusting/inferring the B parameter. Incorporating nontrivial X can contribute and inform the inference of B as well as the free energy of the model. Employing second‐level PEB might allow the identification of survival patterns of progression between the cohort by removing connections that contribute to the complexity of the model. Although this approach may yield finding a model with highest evidence, it arises from a “greedy” search over the model space, including models which may not be clinically plausible. This limits its accuracy in addressing questions of precision medicine.
2. Clinically informed specifying condition‐specific matrix: This sparser CM only allows certain parameters (connections) to change between baseline and annual follow up data, based on those connections that show changes in clinical studies (option 2 in Figure 3). This definition of X informs regions and connections affected by disease progression where subsequently mechanistic modelling by DCM elucidates underlying neuronal causes. This modelling approach excludes connections or regions that are unaffected by disease progression based on current known clinical knowledge. Group‐level model comparison of free energy between this clinically informed DCM and the default option, as explained above, can determine whether our suggested information fusion is useful in terms of model evidence improvement for the given group data. This modelling approach allows one to test hypotheses based on whether clinical information enrich mechanistic models of neuroimaging data. To perform group analysis, we select a set of parameters of interest (e.g., NMDA, AMPA, or between‐regions connections) and then establish a second‐level linear model with PEB to assess commonalities or to associate them with empirical priors (e.g., age or cognitive scores).

## 3 Results

The average source inversion accuracy over the cohort is $R^{2}=89.1$ and individual results are given in the Supporting Information section (Figure S1). The ensuing DMN sources and their interactions are modelled by DCM approach.

### 3.1 Differential BL Versus AF Analysis in DMN Sources

Clinical findings about neurological disorders, particularly Alzheimer's disease (AD), support disconnection hypotheses where some brain regions show lower‐level activity and their interactions with other regions are altered (Kumar et al. 2021). In the context of Alzheimer's disease (AD) progression, the default mode network (DMN) is affected, with the PCC and MPFC expected to show alterations over time for example, (Leech and Sharp 2014; Seoane et al. 2024).

### 3.2 Longitudinal DCM Structure Learning

We inverted from MEG spectral features in the default mode network using DCM, followed by a second‐level analysis to determine the effects of time, between BL and AF datasets. To assess the impact of each modification to DCM, we performed model comparisons within each group and across the entire model space as follows:

#### 3.2.1 Free Energy Comparisons of DCM With Default CM

**FIGURE 4:**
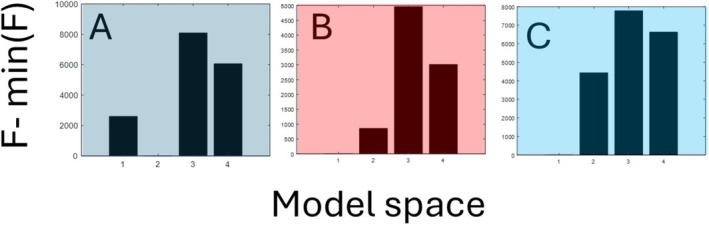
Model comparison between each group of hypotheses about alteration of neuronal mechanisms between baseline and follow‐up data. The first model in each group is default DCM, the second model is DCM where NMDA/AMAP parameters are separately inferred. The third model adds region‐specific STLs, and the fourth model is with a CM. The difference between the panels A–C concerns the way that the condition‐specific matrix CM is defined: (A) default CM; (B) CM informed by clinical hypothesis; (C) CM informed by clinical information, plus differential AMPA/NMDA effects as per 2.2.1.

We compared the free energies associated with group DCMs for: (1) default DCM, (2) DCM with separated AMPA/NMDA parameters, (3) DCM with region‐specific STL parameters, and (4) DCM with both separated AMPA/NMDA parameters and region‐specific STL parameters. In all cases, CM was the default (i.e., X=1). These results are shown in Figure 4A, which reveals that the model with region‐specific STL has the largest free energy. The maximally parametrised model has the second‐highest free energy. Interestingly, in the absence of region‐specific STL, DCM could not disentangle AMPA/NMDA, that is, the second model fared worse than the first. This indicates that simple separation of the glutamate parameters may have adverse consequences for model evidence as the result of increased model complexity.

#### 3.2.2 Free Energy Comparisons of Longitudinal DCM Informed by Disconnection Hypothesis

The four‐model comparisons described in the previous section were repeated, but now with the CM matrix turning off parameters (B connections) that did not show any change based on clinical information (Option 2 in Section 2.2.2.2). The results are shown in Figure 4B. There was a different rank ordering of the four models compared to the default CM in Figure 4A, although the most likely model structure was the same, with individual STLs. Note that in the absence of individual STLs, separated NMDA/AMPA connections did better than non‐separated ones (but neither led to the winning model).

**FIGURE 5:**
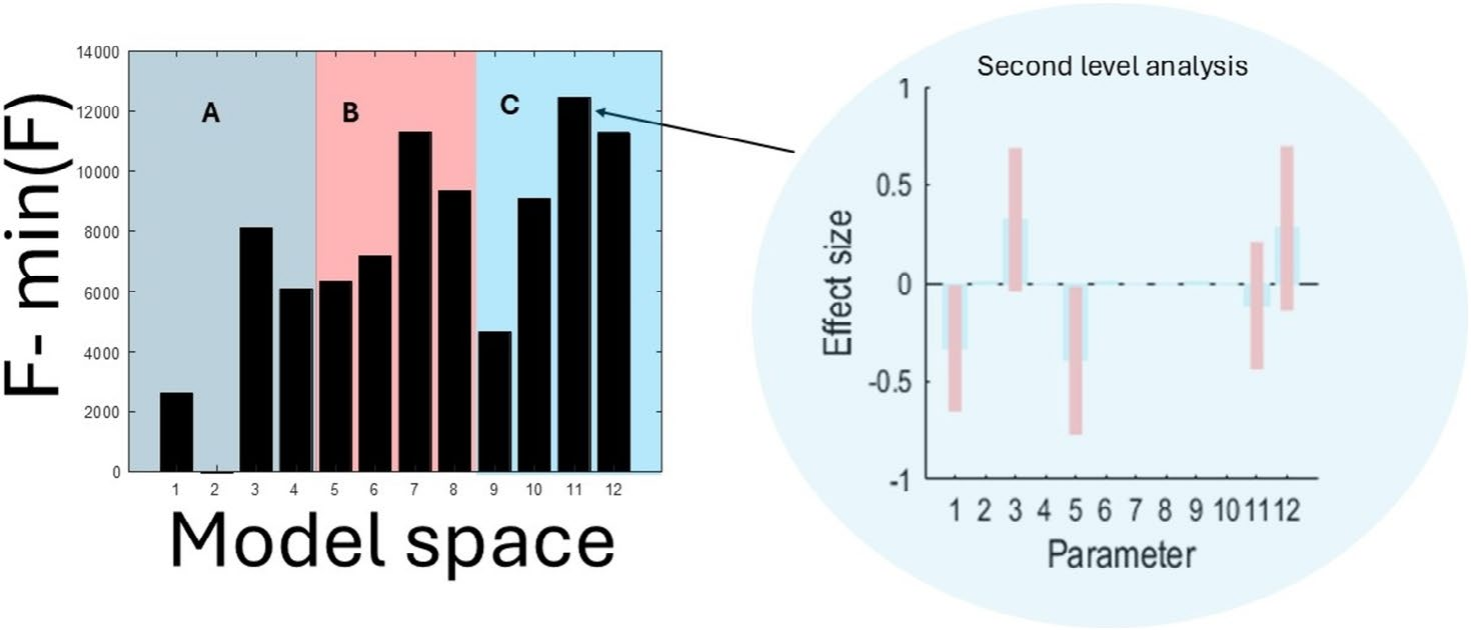
The whole model space, with PEB of winning model followed by Bayesian model averaging by PEB. In the winning model there is a reduction in MPFC activity that is mediated by reduction in superficial/deep layers gains and decrease in AMPA projections between MPFC and LAG and reduction in NMDA links between MPFC and RAG. This contrasts with the increase in PCC activity through changes in superficial/deep gains as well as its NMDA connections to LAG. Five of the connections are retained after PEB and are listed in bold below: The connections 1 to 12 refer to (1) **self‐inhibition of superficial and deep layers in MPFC**, (2) Distal AMPA connections changes between MPFC and PCC, (3) **self‐inhibition of superficial and deep layers in PCC**, (4) Distal AMPA projection between PCC and LAG, (5) **Distal AMPA projection between MPFC and RAG**, (6) Distal AMPA projection between PCC and RAG, (7), intrinsic connections between superficial to spiny cells and deep layers of MFPC, (8) NMDA connections between MFPC and PCC, (9), intrinsic connections between superficial to spiny cells and deep layers of PCC, (10) NMDA connections between PCC and RAG, (11) **NMDA connections between MFPC and RAG** and (12) **NMDA connections between PCC and LAG**. Predicted versus observed responses of each individual DCM are provided in the supplementary information (Figure S2).

Prior information about the effect of AD from postmortem data (Domínguez‐Álvaro et al. 2021) suggests that changes in expression of AMPA and NMDA receptors in layers II, III, and V occur in AD. Therefore, we allowed them to be altered by CM to model the change between BL and AF. Inclusion of this information led to improvement in NMDA/AMPA‐DCM model evidence compared to the default DCM, as shown in Figures 4C and 5.

The winning model provided evidence for selective changes in NMDA receptor‐mediated transmission, and progressive alterations in connectivity between the precuneus and medial prefrontal cortex may be associated with cognitive decline in AD. These findings suggest that AD progression is characterized by disruption of specific neurotransmitter systems within key regions of the DMN, which play critical roles in cognitive functions such as memory and attention.

#### 3.2.3 Bayesian Structure Learning Over Entire Model Space

Comparing model evidence across the entire model space is illustrated in Figure 5, where region‐specific STL parameters are guided by clinical information. In the overall winning model (model number 11th in Figure 5) we see alterations in MPFC and PCC: a reduction in superficial/deep layers gains and a decrease in AMPA projections between MPFC and LAG and a reduction in NMDA links between MPFC and RAG, contrasting with an increase in PCC activity through changes in superficial/deep gains as well as its NMDA connections to LAG.

### 3.3 Second‐Level Analysis With Clinical Scores

We tested whether changes in connectivity parameters within DMN were associated with the degree of cognitive decline during the follow‐up period. This was because participant diagnosis was based on clinical assessment, including a significant and symptomatic episodic memory deficit, plus amyloid biomarker status. The ACE‐R score at baseline and follow‐up was used to measure overall cognitive decline. The ACE‐R clinical scores were used as a covariate in the PEB analysis to test associations between changes in effective connectivity and cognitive decline.

**FIGURE 6:**
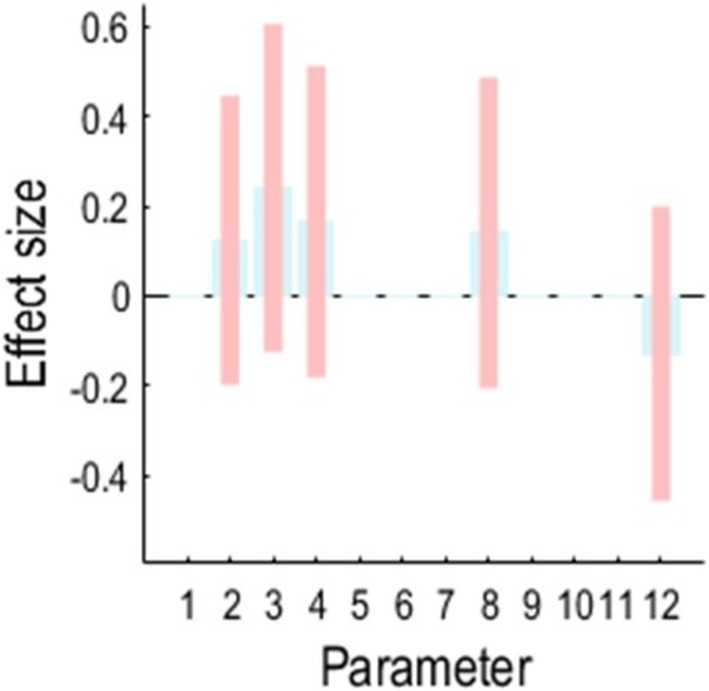
PEB‐based correlates of differences in cognitive decline, and the condition‐specific DCM parameters. The analysis suggested most parameters are directly associated with changes in cognitive decline as AD progressed; expectation is the NMDA connections between PCC and LAG. The connections 1 to 12 refer to (1) self‐inhibition of superficial and deep layers in MPFC, (2) Distal AMPA connections changes between MPFC and PCC, (3) self‐inhibition of superficial and deep layers in PCC, (4) Distal AMPA projection between PCC and LAG, (5) Distal AMPA projection between MPFC and RAG, (6) Distal AMPA projection between PCC and RAG, (7), intrinsic connections between superficial to spiny cells and deep layers of MFPC, (8) NMDA connections between MFPC and PCC, (9), intrinsic connections between superficial to spiny cells and deep layers of PCC, (10) NMDA connections between PCC and RAG, (11) NMDA connections between MFPC and RAG and (12) NMDA connections between PCC and LAG.

We establish PEB with two regressors where the first regressor models the average of connections over the cohort and the second regressor is the Z‐scored differences between annual follow‐up ACER scores and baseline ACER scores to assess the relation between parameters and empirical clinical scores. We repeat the PEB analysis over the aforementioned 12 candidate models. The winning model was again model 11 in Figure 5. The association between differential cognitive scores and parameters is shown in Figure 6. The results indicate that many condition‐specific parameters are directly associated with differential cognitive performance.

This result shows the association between cognitive scores and condition‐specific connectivity parameters. The winning model here shows reduced strength of connections between spiny cells and deep in MPFC and a reduction between spiny cells and deep in MPFC, in relation to cognitive decline. Comparing all models across the hypothesis space confirms that the model informed by clinical information and individual cognitive scores had the highest free energy.

## 4 Discussion

This paper introduces a framework for longitudinal neuronal modelling of Alzheimer's disease progression aimed at revealing the effects of neurological disease in terms of regional, laminar/cell‐class and receptor‐class differences in cortical circuits. We show that the inversion from magnetoencephalography to conductance‐based dynamic causal model, can leverage clinical hypothesis and cognition to explain changes in non‐invasive neurophysiological responses over time. The DCM approach in this study incorporated multiple sources of prior information. Prior values for synaptic physiologies are based on preclinical experimental evidence, such as time constants of synaptic transmission, constrained the biophysical plausibility of our models. Clinical hypotheses about AD, like the selective effects on NMDA receptors, guided our model space. We also used empirical priors derived from participants' cognitive scores (ACE‐R) to inform model inference at the group level to relate neurophysiological changes to cognitive decline. This integration of information to distinguish individual regions and participants goes further than current neuronal modelling methods, motivated by the needs for precision medicine studies.

Neurological disorders often affect different areas of the brain to different degrees, and recognition of this selective vulnerability can be sought by comparing model evidence. To achieve this, while remaining sensitive to group effects, we used a Bayesian approach to conduct a repeated measures longitudinal design, reducing the confounding of longitudinal by cross‐sectional effects. The model with the highest evidence identified the effects of disease progression on a subset of connections and region‐specific changes. The second highest evidence was for a more complex model, suggesting that while increased model complexity can be beneficial in terms of accuracy, additional complexity is penalized. One key improvement in the condition‐specific matrix was the separation of NMDA/AMPA parameters, highlighting the role of informative priors based upon the preclinical evidence of differential biological effects of the disease in question. Region‐specific STL parameters, when informed by clinical hypotheses about the disconnection hypothesis, also improved the generative model of neuroimaging data. Through second‐level analysis with differential cognitive scores as an empirical prior, we confirm neuronal changes between baseline versus annual follow‐up are associated with clinical assays of cognitive decline.

DCM analysis revealed several key findings that have direct relevance to the aetiology and pathophysiology of AD. First, the observed alterations in NMDA receptor‐mediated transmission within the DMN align with the well‐established role of NMDA receptors in synaptic plasticity and cognitive function, particularly in memory and learning (Jobson et al. 2021; Euston et al. 2012; Babaei 2021; Ning et al. 2024; Schoonhoven et al. 2022, 2023). Clinically, these NMDA‐receptor dynamic changes may contribute to the episodic memory deficits that are a hallmark of AD, given that NMDA receptor dysfunction disrupts the synaptic mechanisms underlying memory consolidation and retrieval (Babaei 2021; Ning et al. 2024). Indeed, an uncompetitive antagonist at glutamatergic NMDA receptors, memantine, is standard therapy for AD in many countries. Second, we identified changes in effective connectivity between the precuneus and medial prefrontal cortex (MPFC). The precuneus is a key hub for self‐referential processing and episodic memory retrieval (Jobson et al. 2021; Euston et al. 2012; Mevel et al. 2011). Altered connectivity between the precuneus and MPFC could therefore contribute to the difficulties with autobiographical memory and impaired self‐awareness often reported in AD (Schoonhoven et al. 2022, 2023).

Medial prefrontal cortex (MPFC) and Precuneus (PCC) are the key regions of interest within the DMN for our DCM analysis. The MPFC plays a crucial role in executive function, decision‐making, and both past and future temporal projection (Jobson et al. 2021; Euston et al. 2012; Xu et al. 2019). Previous studies have demonstrated reduced activity and connectivity in the MPFC in AD patients using fMRI (Jobson et al. 2021). MPFC is particularly vulnerable to early amyloid deposition and tau pathology in AD (Euston et al. 2012, Xu et al. 2019). Similarly, the Precuneus is a central hub for self‐referential processing, episodic memory retrieval, and visuospatial imagery (Lee et al. 2020; Leech and Sharp 2014; Leech and Smallwood 2019; Fransson and Marrelec 2008) which has shown reduced activity and gray matter volume in AD patients (Palesi et al. 2012). In addition, the Precuneus is one of the first extra‐temporal regions to exhibit amyloid accumulation and metabolic dysfunction in AD (Yokoi et al. 2018; Fransson and Marrelec 2008).

DCM analysis in this study confirmed the reduction in MPFC activity, but identified this as being driven by the gains in superficial/deep layers and decreases in distal AMPA projections between MPFC and LAG, with a reduction in NMDA‐mediated links between MPFC and RAG. This contrasts with the increase in PCC activity through changes in superficial/deep gains and long‐range NMDA‐mediated connections to LAG. These results are consistent with previous studies (Jobson et al. 2021, Euston et al. 2012, Babaei 2021, Ning et al. 2024, Schoonhoven et al. 2022, 2023), but DCM for MEG allows one to go beyond mere correlational findings by inferring the underlying causal neuronal mechanisms. DCM alone cannot identify the upstream mechanisms leading to the disruption of NMDA receptors—such as aggregated beta‐amyloid and phosphorylated‐tau. It can however reveal targetable mediators of cognitive change through the generators of physiological activity and connectivity that underlie cognition and the clinical AD symptoms observed in people with AD.

Our DCM analysis revealed several key findings that have direct relevance to the clinical presentation of AD. First, the observed alterations in NMDA receptor‐mediated transmission within the DMN align with the well‐established role of NMDA receptors in synaptic plasticity and cognitive function, particularly in memory and learning (e.g., Babaei 2021; Ning et al. 2024). Clinically, these findings may help explain the episodic memory deficits that are a hallmark of AD, as NMDA receptor dysfunction can disrupt the synaptic mechanisms underlying memory consolidation and retrieval.

There are limitations to the study. We modeled the interactions between four cortical DMN sources and assume minimal crosstalk in estimating their activity from the sensor data. We did not include some of the regions that are closely associated with Alzheimer's disease, such as the hippocampus and entorhinal cortex. Their exclusion was partly because of the limited sensitivity of external array magnetoencephalography to deep structures like the medial temporal lobe. Specialist procedures, tightly constrained individualized forward models, and prolonged scanning can increase the detection of hippocampal signals (Mccormick et al. 2020), but would be challenging to implement at scale in the context of people with dementia. Despite this limitation, the parsimonious generative model for resting state data derived from the DMN indicated relevant cortical mechanisms by which AD changes the oscillatory brain dynamics over time. Even without medial temporal lobe structures, the regions of the DMN, including Precuneus, Medial Prefrontal Cortex, and Angular gyrus, have extensive evidence of pathophysiological change in AD.

A further limitation is that we did not integrate individual data on tau, amyloid, or synaptic density changes for example from PET scanning (Ossenkoppele et al. 2018; Schoonhoven et al. 2023; van der Kant et al. 2020; Kocagoncu et al. 2020; Venkataraman et al. 2021). A single modality analysis of neuronal dynamics is a potential limitation, but MEG data was sufficient for neuronal estimation and Bayesian model comparison over the cohort. However, additional hypotheses regarding human disease progression and treatment to slow progression could be tested by considering MEG together in conjunction with other modalities (Adams et al. 2023; Jafarian et al. 2023, 2021). Finally, akin to all modeling practices, even those supported by the highest free energy, careful consideration of parameter separation and integration of empirical priors is essential for meaningful interpretation of model performance.

We focus on changes within a patient group, and do not compare them to the trajectory in healthy controls. This anticipates the type of design in future clinical studies of progression or clinical trials of experimental treatment. Future studies could also address longitudinal ageing, with the same methodology. Our longitudinal study design also had just two time‐points; resting‐state MEG protocols can be readily and safely repeated, making them suitable for serial assessments.

Future studies may adopt whole‐brain approaches and multi‐modal data integration. Building on our demonstration of DCM's reliability in tracking disease progression, we envision several potential applications. These include predicting individual treatment responses based on synaptic dynamics profiles, personalized monitoring of treatment efficacy through real‐time tracking of synaptic changes, earlier characterization of disease mechanisms as presymptomatic alterations in synaptic function, and improved insights from early‐phase clinical trials.

Our approach to DCM offers several routes to facilitate early phase clinical trials, including AD. First, by determining specific synaptic mechanisms associated with a given disease, its severity and progression, DCM outputs could prioritize the selection of targets for novel therapies. Second, patients may be stratified by inversion from their observed physiology (MEG, or EEG) to subject‐specific and region‐specific generative mechanisms for that physiology, identifying subgroups who may respond preferentially to treatment. Third, DCM outputs can provide sensitive, reliable, and objective measures, such as surrogate or intermediate outcomes of treatment. Finally, DCM could be used to enrich trial populations by selecting participants who exhibit the most relevant neurophysiological characteristics or changes in the pharmacological target of the drug in question.

In conclusion, we have shown that enhanced dynamic causal models of non‐invasive brain imaging confirm the predicted mechanisms of change underlying Alzheimer's disease progression. There are three key contributions, which extend to modelling of other disorders: First, the inclusion of regional inhomogeneity of the contributions of neuronal cells to the mean field observations, respecting regional variance in disease burden; second, the dual parameterization of excitatory neurotransmissions, motivated by preclinical and clinical evidence of distinct effects of disease on AMPA versus NMDA type glutamate receptors; and third, the inclusion of individualized condition effects, for modelling the effects of individual responses to disease progression and for drug response in future intervention studies. We hope that these methods will facilitate early‐phase human trials for the development and assessment of much needed new therapeutics.

## Author Contributions

**Amirhossein Jafarian:** conceptualization, methodology, software, validation, formal analysis, writing original draft, data curation, visualization. **Melek Karadag Assem:** data acquisition, pre‐processing of MEG data, review and editing. **Ece Kocagoncu:** data acquisition, pre‐processing of MEG data, review. **Juliette H. Lanskey:** data acquisition, review. **Haddy Fye:** programme manager, review and editing. **Rebecca Williams:** discussion, review and editing. **Andrew J. Quinn:** data acquisition, review. **Jemma Pitt:** data acquisition, review. **Stephen Lowe:** review. **Vanessa Raymont:** review. **Krish D. Singh:** review. **Mark Woolrich:** review. **Anna C. Nobre:** discussion, review and editing. **Richard N. Henson:** discussion, review and editing. **Karl J. Friston:** discussion, review and editing. **James B. Rowe:** conceptualization, methodology, review and editing, funding acquisition.

## Conflicts of Interest

Mark Woolrich is a member of the HBM Editorial Board and co‐author of this article. The other authors declare no conflicts of interest.

## Supporting information


**Figure S1.** R2 statistics of source inversions amongst all subjects.
**Figure S2.** Observed baseline and follow up data and their predicted dynamic causal modelling results for each individual subject.

## Data Availability

The data that support the findings of this study can be requested from the senior author, noting that a data transfer agreement may be required under the terms of consent and data protection legislation. Anonymised data will also become available via https://www.dementiasplatform.uk/data‐portal. The code associated with this manuscript is available at https://github.com/NIMG‐22‐2183/L‐DCM_HBM. The SPM12 software is openly aviable at https://www.fil.ion.ucl.ac.uk/spm/.

## Appendix A Principal of Dynamic Causal Modelling

DCM was developed to infer biological causes of neuroimaging data (e.g., fMRI, M/EEG, MEG‐fMRI) (Jafarian et al. 2019, 2020). Unlike other techniques such as structural connectivity analysis, which is used to explore the presence of axonal connections between brain regions, and functional connectivity analysis, which entails measuring statistical dependencies between sources, DCM quantifies causal and directional influences (i.e., effective connectivity) for hypothesis testing. DCM can be used to specify, infer, and compare multiple hypotheses from neuroimaging data, and select the most likely one that defines causal influences between/within brain regions.

Model inference in DCM is based on optimizing free energy using a variational Bayes method to estimate synaptic physiology from empirical data (Friston et al. 2007). There are two critical aspects to using free energy for inference in this way. Firstly, it effectively prevents overfitting. Secondly, Free Energy facilitates comparing different models through Bayesian model comparison. The outcome of DCM inference includes predicted responses and posterior distributions of its parameters. The free energy estimate (lower‐bound on the model evidence) is a quantity that balances accuracy and model complexity. DCM optimizes the free energy (with respect to all unknown model parameters) to infer parameters that accurately generate the data and are not “too complex”. This decreases the chance of overfitting. To compare different models, we utilize the Bayes factor, which is the ratio of the free energies under two competing models of the data. One thereby determines which hypothesis is more likely given the observed data.

A key DCM principle in modelling different data features is that neuronal dynamics regress to a stable equilibrium in the absence of any input (Jafarian et al. 2021). Different types of inputs induce time/frequency domain data features. For example, experimental inputs like visual or auditory stimuli lead to event‐related potential (ERP), or random neuronal fluctuations induce oscillations around the neuronal equilibrium. In both cases, we aim to incorporate these key mechanisms to facilitate the inversion of biological models (Adams et al. 2023; Jafarian et al. 2023).

In this paper, we used a conductance‐based neural model, based on the Morris‐Lecar simplified Hodgkin‐Huxley model (Jafarian et al. 2024, 2023). These models incorporate detailed physiology as shown in Figure 1, where the dynamics of membrane potentials include several ion currents, such as NMDA, AMPA, and GABA. The rate of conductance changes is scaled by input firing rates, meaning that presynaptic inputs directly influence synaptic response in the target population. The laminar structure of this model encompasses superficial and deep pyramidal cells, representing the dynamics of neurons in superficial and deep layers of cortical columns, respectively. It also includes spiny stellate excitatory cells in layer IV and an inhibitory interneuron population within the cortical column. These populations are interconnected via intrinsic excitatory or inhibitory connections, informed by anatomical connections observed in the brain. There are also top‐down and bottom‐up extrinsic connections between regions that interconnect cortical columns. Forward connections are from superficial pyramidal cells to spiny stellate and deep layers of higher‐level brain hierarchy. Backward connections, on the other hand, are from deep pyramidal cells back to inhibitory and superficial layers of lower anatomical regions. External inputs primarily target spiny stellate cells and often originate from the thalamus.

The local field potential generated by a cortical column in DCM is the activity of the superficial layers, plus a smaller‐weighted contribution of the activity of the deep and excitatory cells, which is estimated from data. The summed activity is scaled by the sensor gain or lead field (i.e., forward electromagnetic head model) to generate LFP or M/EEG data (Jafarian et al. 2024).

DCM relies on Bayesian inversion, in which well‐defined priors are crucial (Jafarian et al. 2024, 2020, 2021; Adams et al. 2023; Adams, Pinotsis, et al. 2021; Cope et al. 2022; Lanskey et al. 2024), in particular to ensure that the system can reach a stable equilibrium. In addition, the parameterisation of the model has a significant impact on model complexity and, consequently, model evidence. Therefore, several approaches like multimodal DCM with MRS and PET have been developed to reduce the complexity of posterior estimates of parameters and to reduce collinearity between parameters. These can inform the biological interpretation of the optimal model.
