# Supplementary material for: Neurophysiological Progression in Alzheimer's Disease: Insights From Dynamic Causal Modelling of Longitudinal Magnetoencephalography
Source: Hum Brain Mapp. 2025 May 21;46(8):e70234. doi: 10.1002/hbm.70234 (PMC12093352; doi:10.1002/hbm.70234)
Supplement: Supplementary file 1 — Figure S1. R2 statistics of source inversions amongst all subjects. Figure S2. Observed baseline and follow up data and their predicted dynamic causal modelling results for each individual subject. [file HBM-46-e70234-s001.docx]

# Supplementary information:

The R2 statistics of the source inversions of resting state eyes open amongst all subjects is shown in supplementary Figure 1.


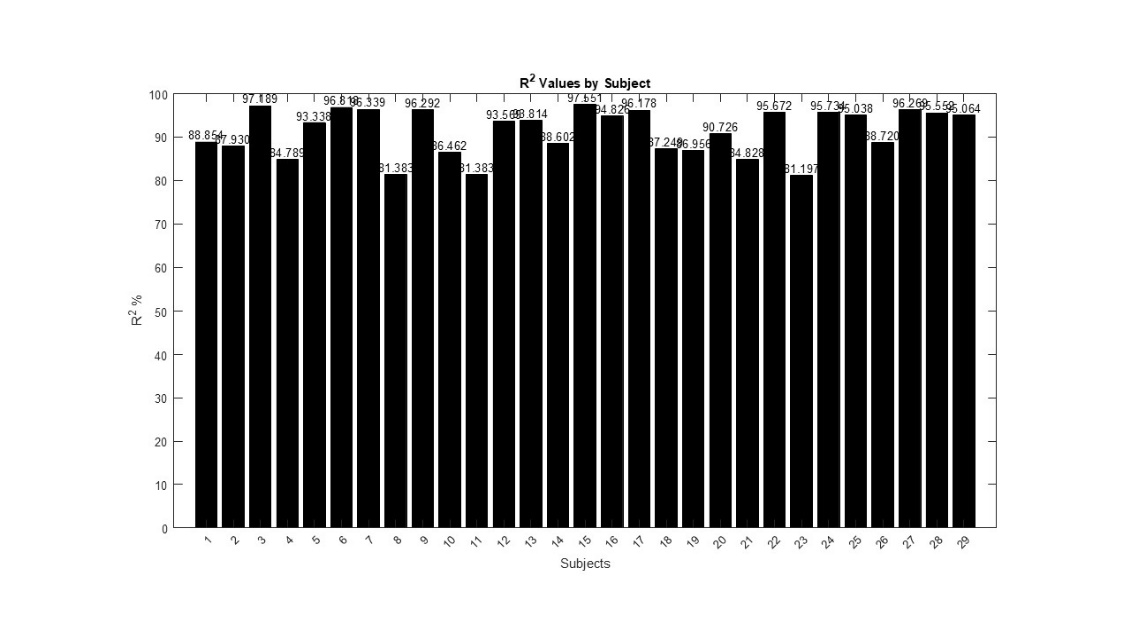


Supplementary figure 1. R2 statistics of source inversions amongst all subjects.

The following graphics (Supplementary figure 2) represent observed power spectral densities of four nodes default model network at baseline and one year follow up, and their predicted DCMs inversion results.


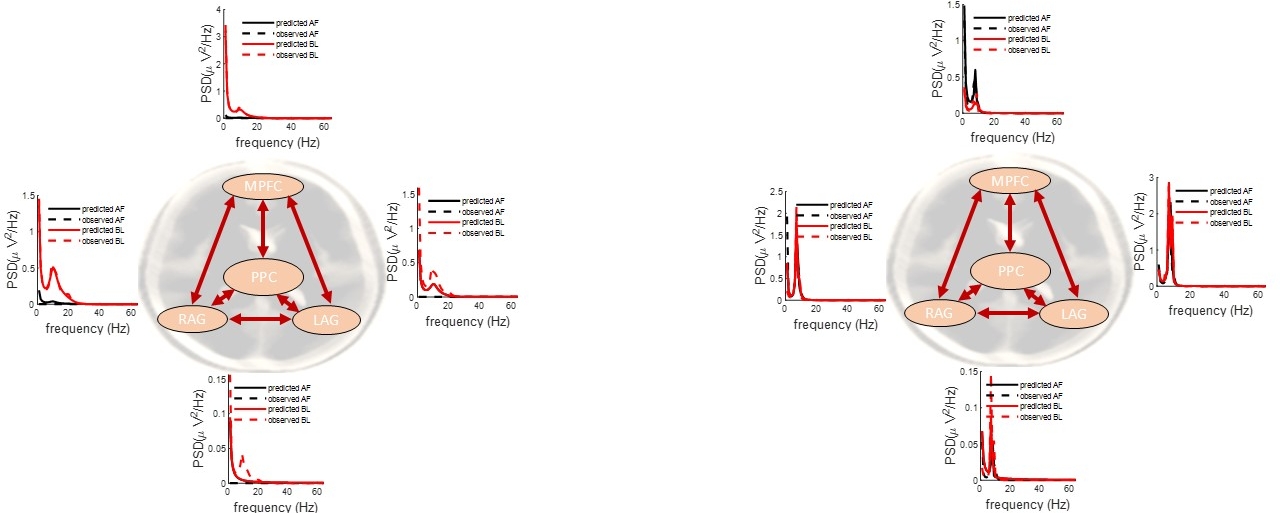


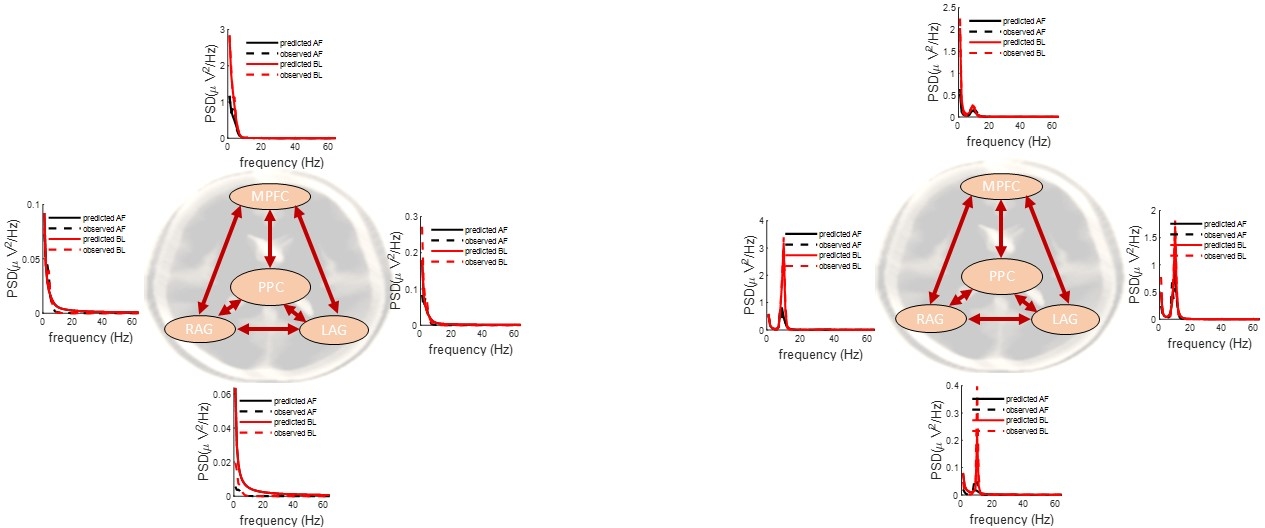

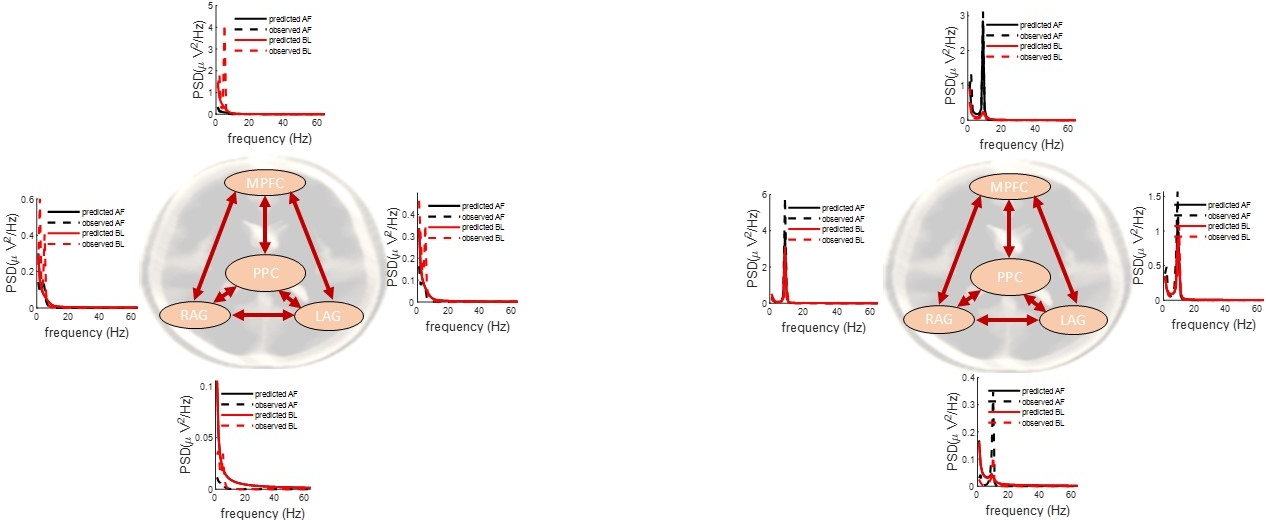

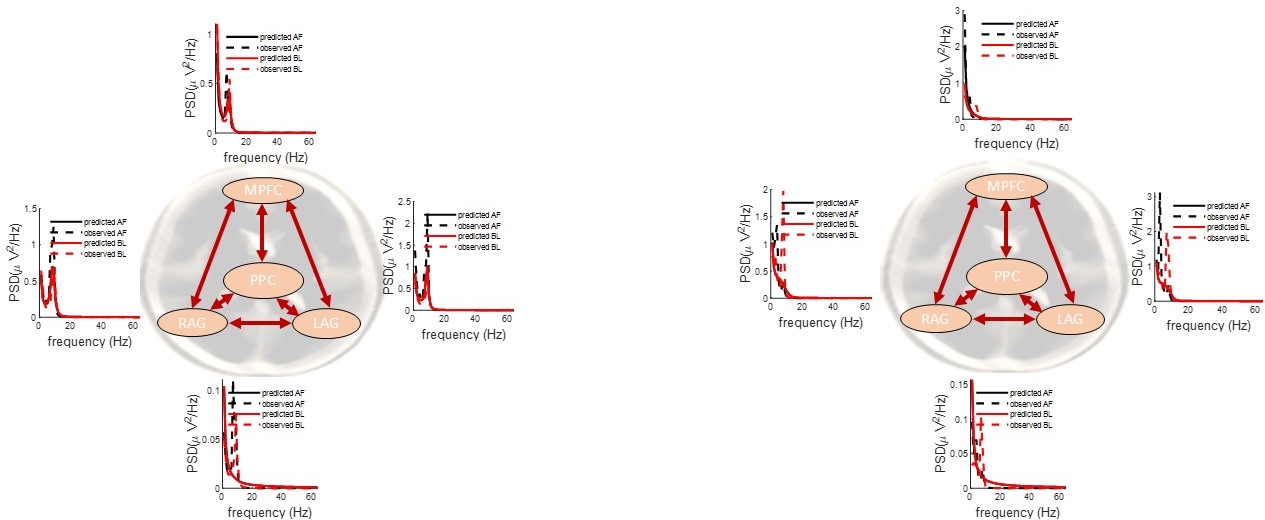

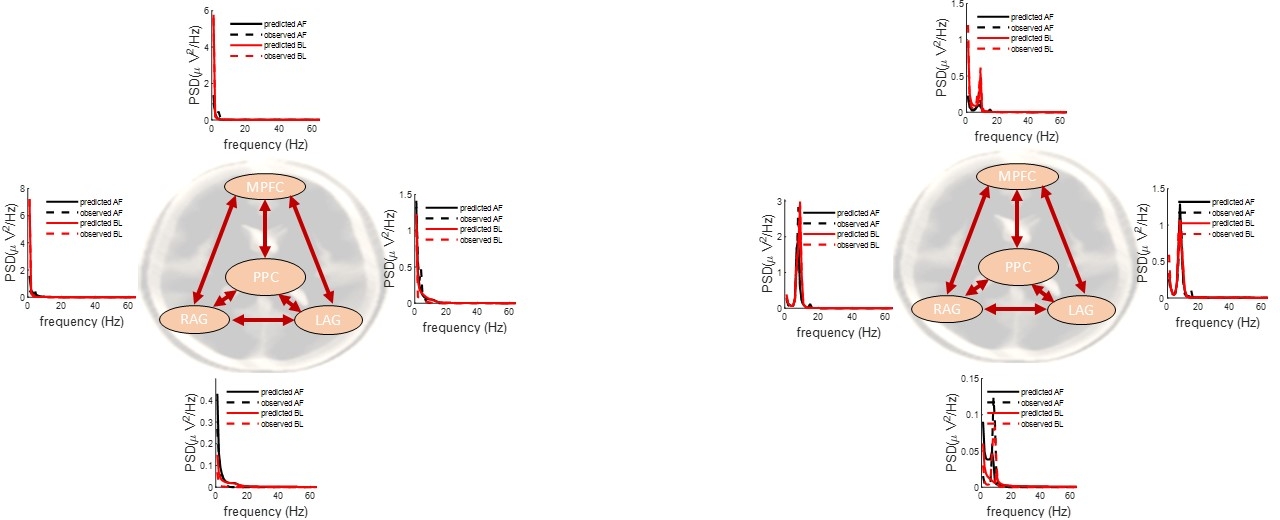

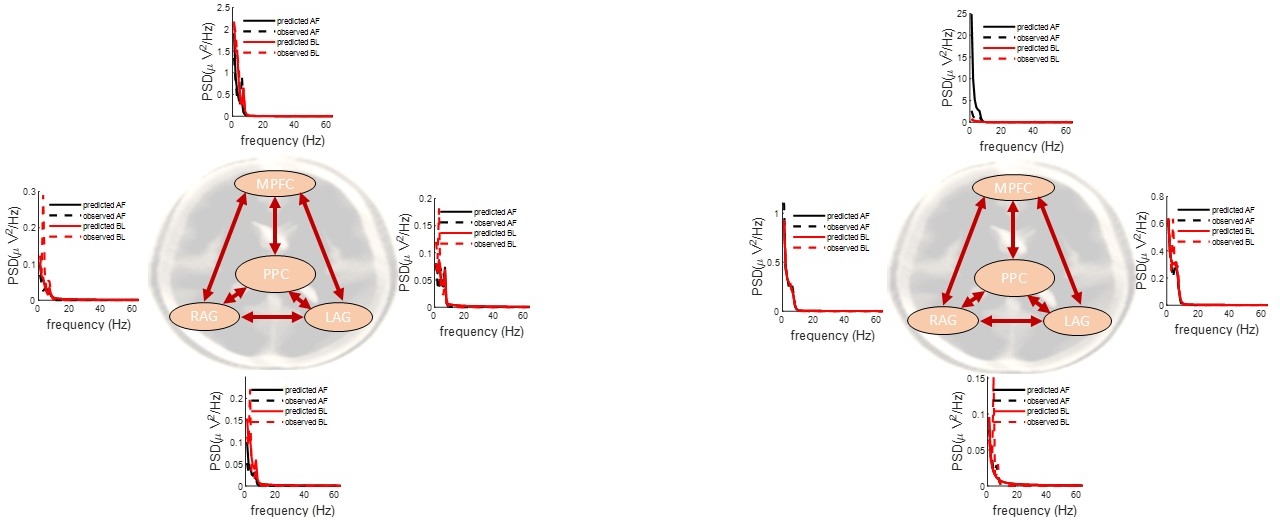

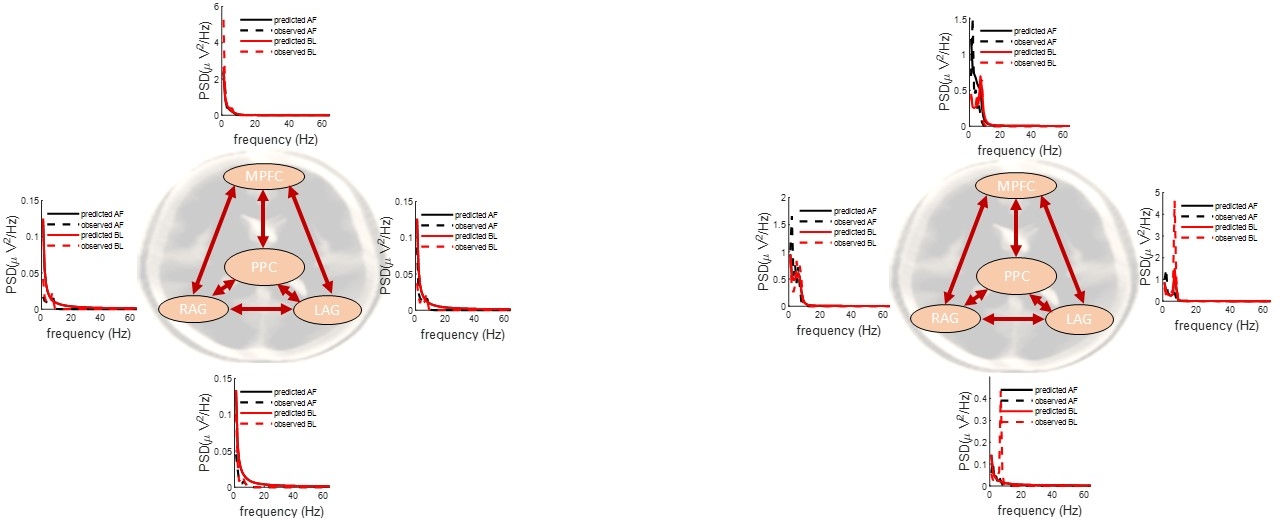

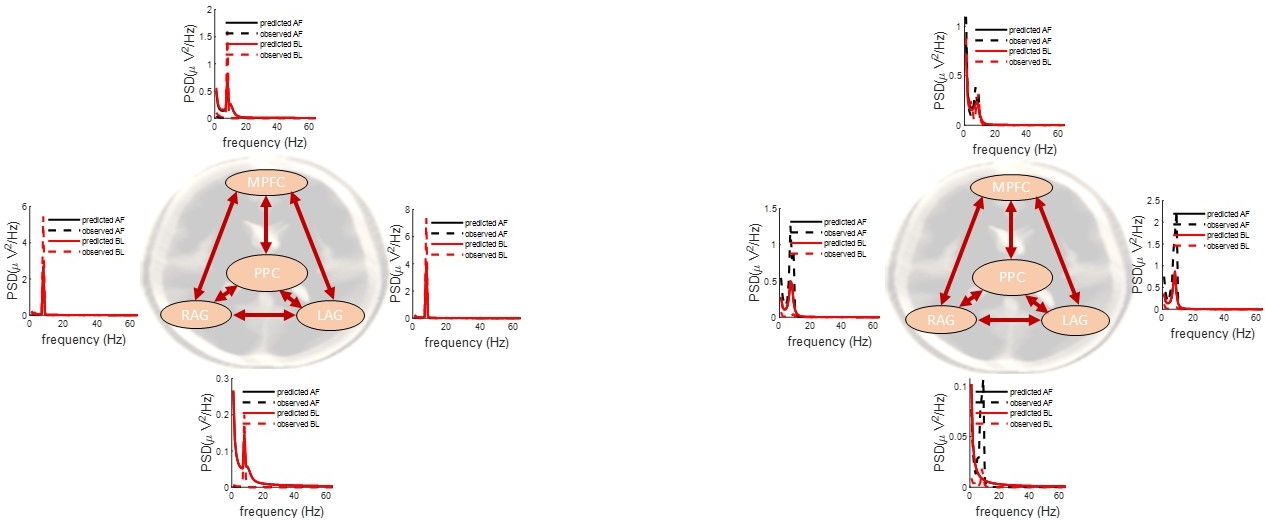

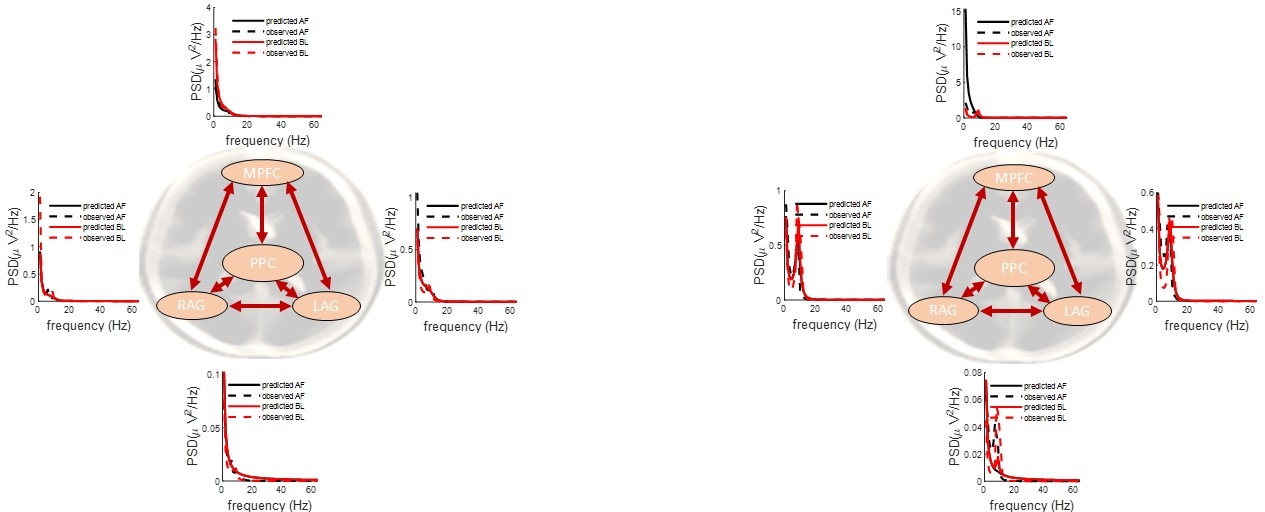

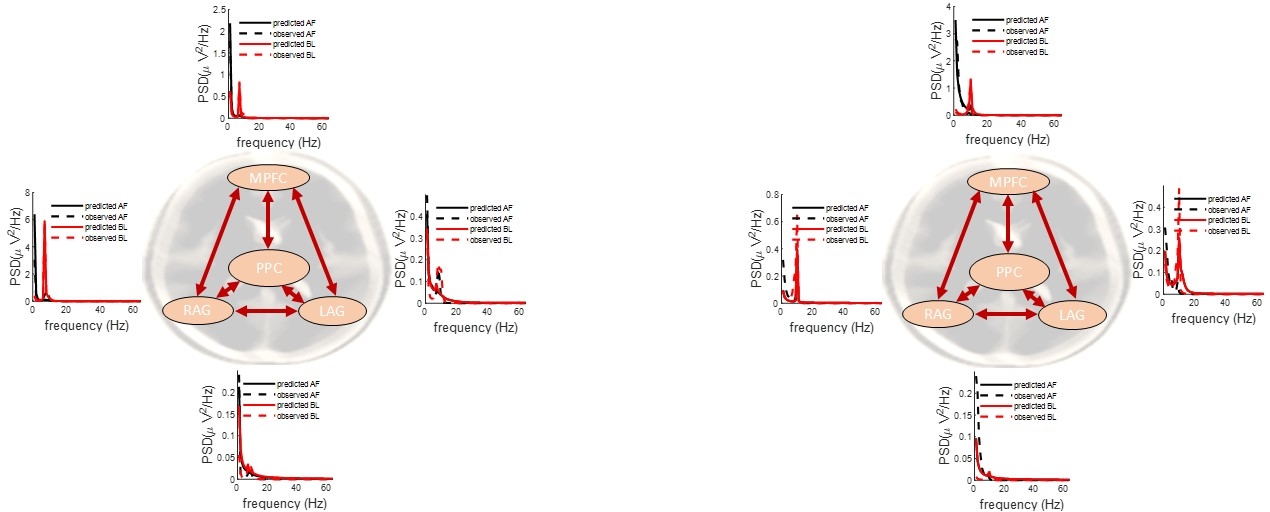

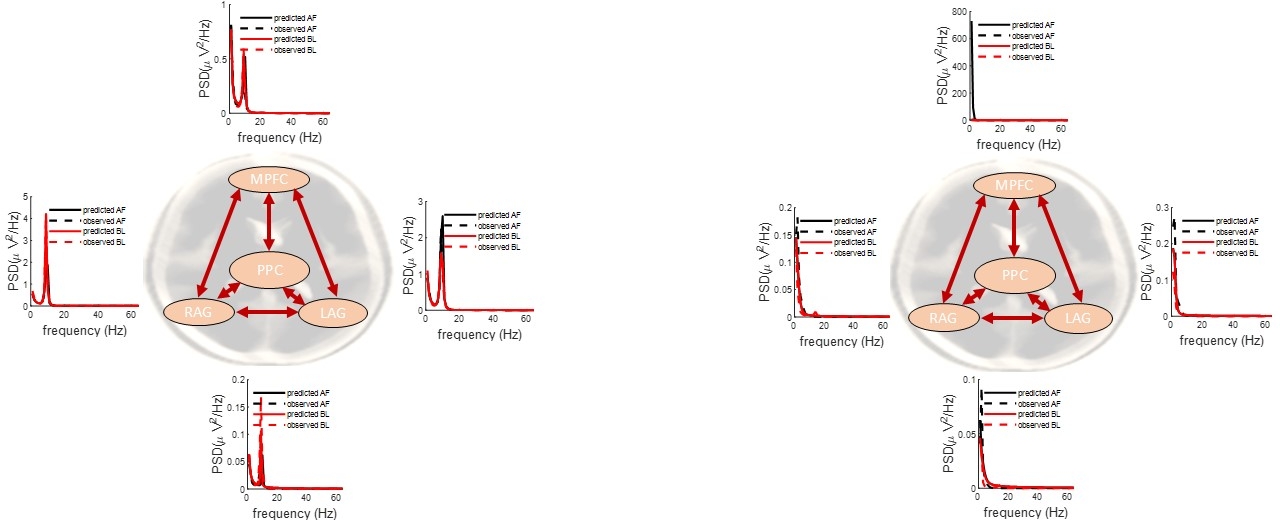

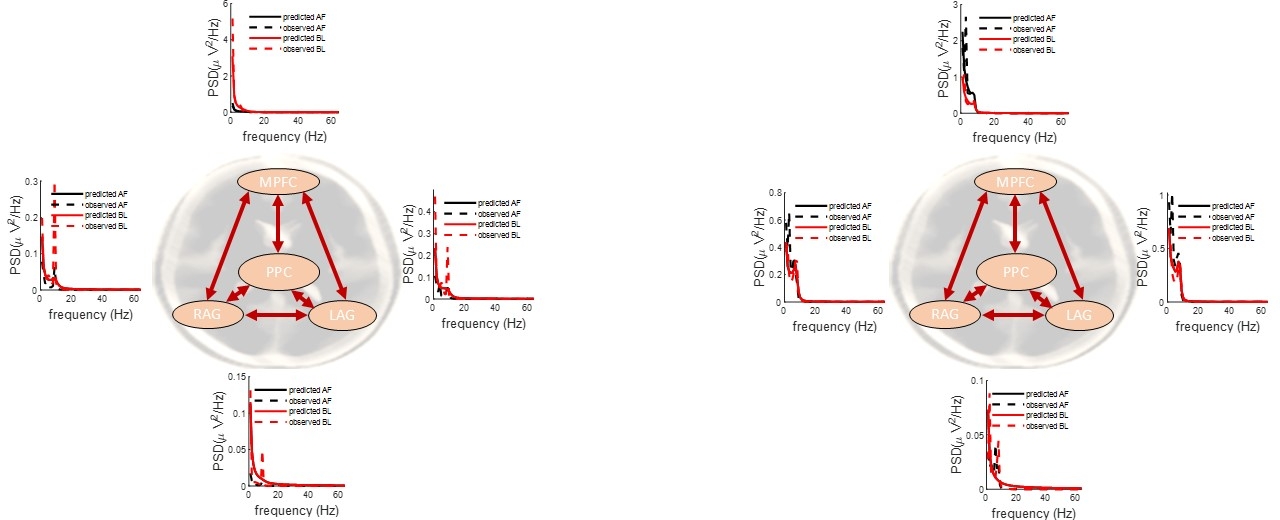

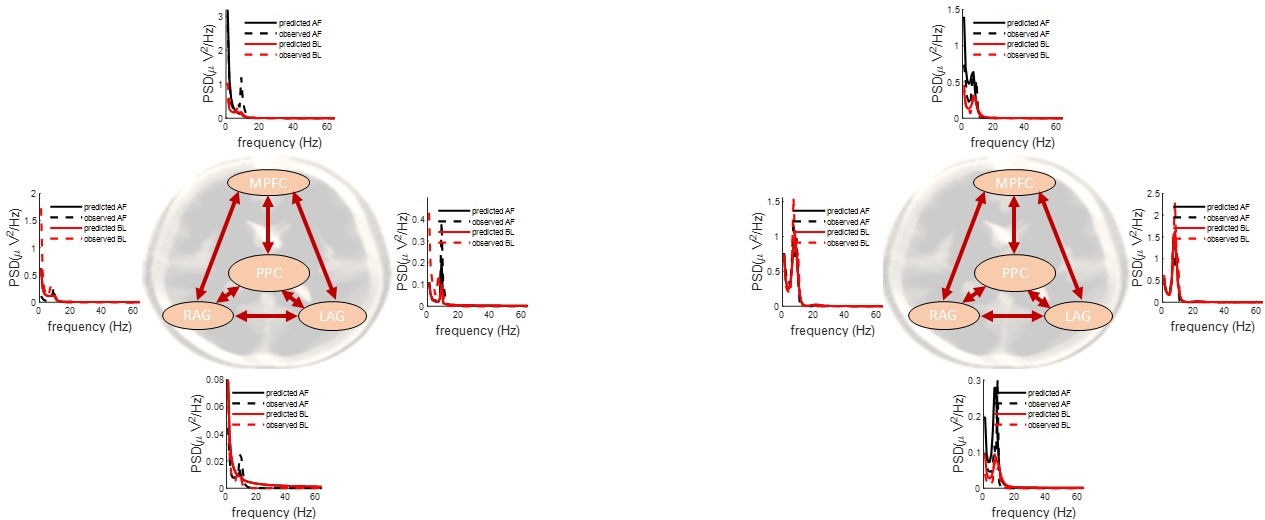

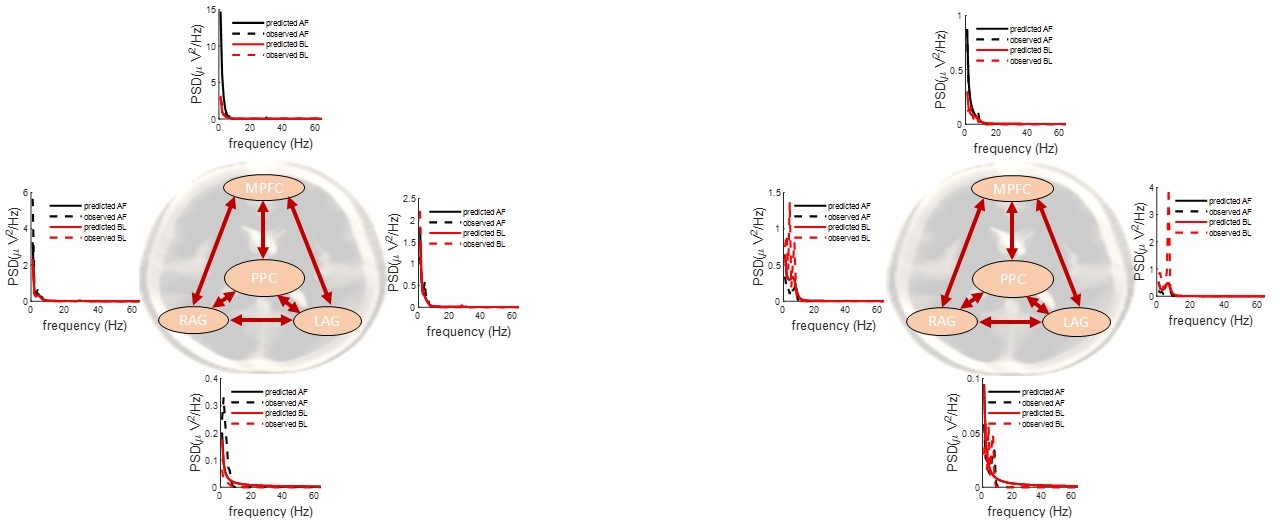

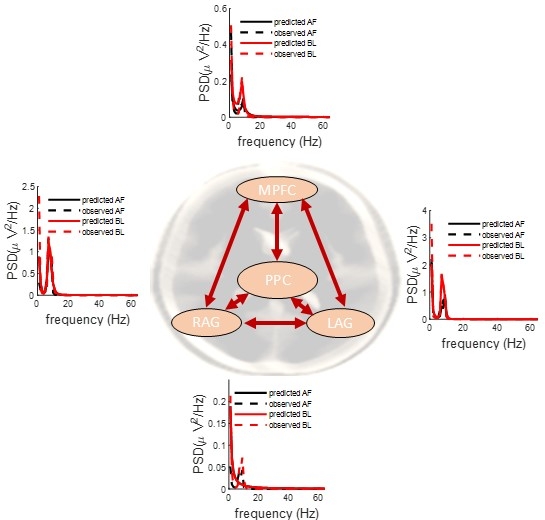


Supplementary figure 2. Observed baseline and follow up data and their predicted dynamic causal modelling results for each individual subject.
